# A Novel Religious/Spiritual Group Psychotherapy Reduces Depressive Symptoms in a Randomized Clinical Trial

**DOI:** 10.1007/s10943-015-0113-7

**Published:** 2015-08-30

**Authors:** Yoichi Chida, Stephanie Schrempft, Andrew Steptoe

**Affiliations:** Faculty of Human Happiness, Happy Science University, 4427-1 Hitomatsuhei, Chosei-mura, Chosei-gun, Chiba 299-4325 Japan; Department of Medical Science, Happy Smile Clinic, Kawasaki, Japan; Department of Epidemiology and Public Health, University College London, London, UK

**Keywords:** Buddhism, Causality, Happy Science, Reincarnation, Spiritual group psychotherapy

## Abstract

This randomized controlled trial aimed to examine the effect of the Happy Science doctrine-based group psychotherapy on depressive symptoms in 118 Japanese mental disorder outpatients. The treatment group (*n* = 58) took part in five 90-min sessions at one-week intervals, while the control group (*n* = 60) received standard care including medication. Depressive symptoms were assessed before the intervention, 5 weeks after the intervention, and at 3-month follow-up. Compared to the control group, the treatment group showed a significant reduction in depressive symptoms both at post-intervention and at 3-month follow-up. In conclusion, this group psychotherapy might be of benefit in treating depressive symptoms.

## Introduction

*Religiosity* and *spirituality* can be defined broadly as any feelings, thoughts, experiences, and behaviors that arise from a search for the “sacred,” with the former implying group or social practices and doctrines and the latter tending to refer to personal experiences and beliefs (Hill et al. 2000). There is an extensive literature relating religiosity/spirituality with mental and physical health (Koenig et al. 2012). For example, a meta-analysis of 147 studies demonstrated a robust but modest (*r* = −0.096) negative correlation between religiosity/spirituality and depressive symptoms, due in part to favorable effects on coping with stressful life events (Smith et al. 2003). Furthermore, we meta-analyzed the longitudinal associations between religiosity/spirituality and mortality from 1960 to 2008, reviewing sixty-nine studies in healthy populations (Chida et al. 2009). We concluded that religiosity/spirituality reduced the odds of dying by 18 % (HR 0.82, 95 % CI 0.76–0.87, *p* < 0.001 controlling for potential confounders).

Regarding religious and/or spiritual psychotherapy (e.g., religious forgiveness, spiritual coping therapy, spiritual history taking, spiritual teaching program, meditation, distant intercessory prayer), a number of clinical trials have been tested on patients with psychiatric or physical conditions (Koenig et al. 2012). There is, however, less research on the use of more specific Eastern spiritual techniques, such as Buddhism, in psychotherapeutic practice (Kelly 2008). In a previous study (Chida and Kim 2011), we developed a novel religious/spiritual group psychotherapy (called “Happy Smile Clinic (HSC) group psychotherapy”) based on Buddhist philosophy.

### Principles Underlying HSC Psychotherapy

Two major features of Buddhism are first, that the nature of the soul is divine, and that if you perceive the law of “Causality” (i.e., cause and effect) and lead the best possible life in accordance with this law, then you can progress toward a being of higher spiritual level (Allen 2006; Okawa 2004). Second, in connection with any discussion of the meaning of cause and effect, the idea of “Reincarnation” is very important. Indeed, reincarnation has also been incorporated into some recent Western psychiatric practices such as Past-Life therapy (Weiss and Weiss 2012).

In addition to these Buddhist principles, the HSC group psychotherapy includes the Principles of Happiness of “Fourfold Paths” preached in Happy Science which is one of the largest Japanese religious institutions (Okawa 2009, 2013; Kurokawa et al. 2015). The first principle of Fourfold Paths is the principle of Love. When we give love, we discover that our thirst for love and happiness is eased. Love is also to believe in other people, recognizing that we are all children of God. As such, we have to suppress some of our less admirable desires, and instead, be kind to those around us and try to live a life that is of benefit to them and to society as a whole.

The second principle is the principle of Wisdom. Wisdom means deep, life-knowledge and includes knowing spiritual facts and the Universal Truth. Knowledge is power. It provides us with the answers to solve our problems and relieve us from our worries. Furthermore, by knowing the Truth, we can transform our lives by turning our former problems into rewarding experience. In turn, the experience will allow us to speak words of wisdom to guide others. The HSC group psychotherapy makes wisdom available to patients to encourage a shift from “Perfectionism” toward “Optimalism.” Optimalism is a philosophical form of optimism, which specifically refers to a belief that while the world may not be perfect it is better than the available alternatives (Ben-Shahar 2009). The Optimalist understands that failures in life are part of the learning process and appreciates the journey toward success. In contrast, the Perfectionist rejects the limits of human ability, and is driven by the fear of failure (Ben-Shahar 2009). This all-or-nothing approach leads to procrastination and, more generally, to inefficient use of time.

The third principle is the principle of Self-reflection. We think that once a mistake is made, it is fixed forever in reality and cannot be altered. In a material sense, this is true. But what happens in the mind encompasses past, present, and future. When we regret and truly repent on our actions, the mistake can not only be undone, but the blessings we receive for awakening the Truth will far outweigh what we had before. Genuine self-reflection can offer far more than simple forgiveness and provide support, encouragement, power, and energy.

The fourth principle is the principle of Progress. There are many methods to win or succeed whether in business or in life. The law of thought is that both good and bad thoughts will someday be manifested. By mastering love, wisdom, and self-reflection, we can find ourselves on the right path to progress: one in which the achievement of our goal will contribute to the happiness of all humankind. Optimism and hopefulness have critical effects on the ability to make progress as they protect against depression and promote better physical health (Chida and Steptoe 2008).

### The Present Study

There is a need for more robust trials of HSC group psychotherapy. Our first randomized controlled trial (Chida and Kim 2011) had a very small sample size (26 outpatients), and although we found an effect of the HSC group psychotherapy on depressive symptoms, we assessed outcome effectiveness only immediately after the intervention. Moreover, we did not evaluate baseline religious demographic characteristics which may be powerful confounders especially in trials of religious/spiritual psychotherapy. The present trial aimed to enroll a larger sample size (140 outpatients), to evaluate outcome effectiveness not only at post-intervention but also at 3-month follow-up, and to explore the relationship of religious demographic characteristics with responses to the psychotherapy.

## Methods

### Design and Study Participants

The study used a two-group (intervention vs. a usual care delayed-treatment control) by three-time (assessment at pre-intervention, post-intervention, and 3 months after intervention) trial design and was conducted at the Happy Smile Clinic in Kawasaki city, Kanagawa, Japan, between January 2012 and December 2013.

**Fig. 1:**
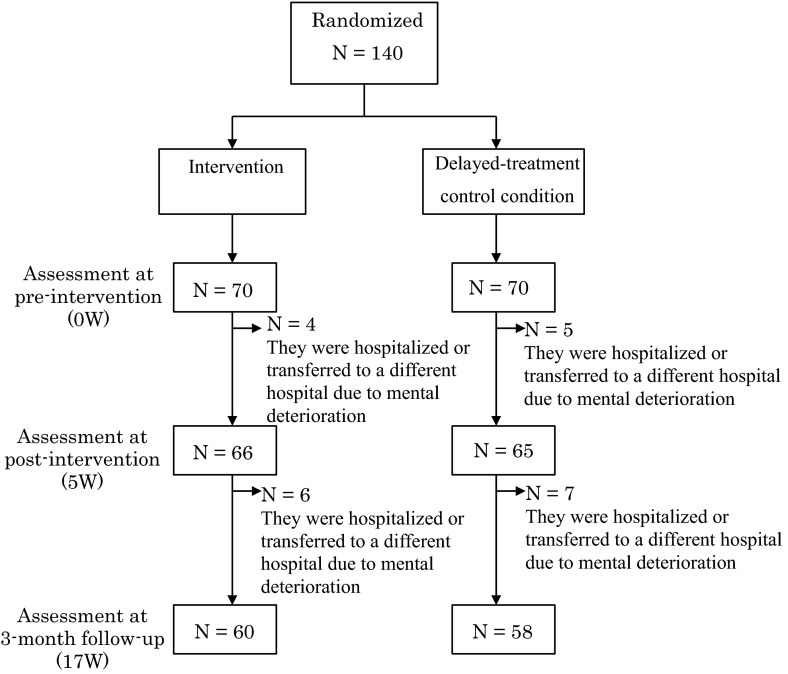
CONSORT flowchart illustrating the recruitment of patients for the present randomized controlled trial

Criteria for enrollment were (1) 18 years or older; (2) Japanese literate; (3) diagnosed with a mood disorder (ICD-10 F30-39), neurotic stress-related and somatoform disorder (ICD-10 F40-48), or eating disorder (ICD-10 F50); (4) no psychotic symptoms; (5) no other medical condition that precluded completion of the self-report questionnaire without assistance. Participants gave full informed consent to participate in the study, and ethical approval was obtained from the Ethics Committee of the Department of Medical Science, Happy Smile Clinic. A total of 140 eligible patients were enrolled and randomly assigned to the intervention (*n* = 70) or delayed-treatment control condition (*n* = 70) (see Fig. 1 for details of patient recruitment). Twenty-two patients (10 in the treatment group and 12 in the control group) dropped out of the study because they were hospitalized or transferred to a different hospital due to mental deterioration, so the dropout rate for the current trial was 15.7 %. There were no significant differences in the demographic characteristics between patients who dropped out and remained in the trial.

### Procedures and HSC Group Psychotherapy

Prior to the intervention, participants completed a set of questionnaires and a written consent form at the clinic. The questionnaires included questions on years of education, marital status, smoking history, alcohol consumption, religious belief, and illness duration. Their body height and weight were measured by nurses at the clinic. Their mental status was assessed at pre-intervention, post-intervention, and 3-month follow-up using the center for epidemiologic studies depression (CES-D) scale Japanese version, which has been validated previously (Shima et al. 1985). The CES-D scale consists of 20 items and assesses total depression symptoms together with four subscales: depressed affect, positive affect, somatic complaints, and interpersonal difficulties (Radloff 1977).

As described previously (Chida and Kim 2011), the 5-week (90 min/week) HSC group intervention (Table 1) consisted of orientation to symptoms from the view of Spiritual life lessons; especially Causality and Reincarnation (Session 1), and skills on how to shift from “a Love that takes” toward “a Love that gives” (Session 2), from “Perfectionism” toward “Optimalism” (Session 3), from “Anger, hatred, and grudge” toward “Forgiveness” (Session 4), and from “Hopelessness” toward “Hopefulness” (Session 5). Session 1 has patients who learn the view of Spiritual life by introducing some recent Western psychiatric practices such as Past-Life therapy (Weiss and Weiss 2012). Patients are encouraged to recognize that their current problems reveal what kind of challenges they have to overcome in this life, and that they are currently experiencing a crucial time for their spiritual growth. Session 2 encourages an appreciation of “love that gives” by asking patients to recall situations where they had been taken care of by others. Session 3 uses the Pareto principle (also known as the 80/20 rule) (Koch 2005) to encourage a shift from Perfectionism toward Optimalism. The patients are instructed to create a list of their real-life problems, order them in terms of priority, and adopt the 80/20 rule whereby they invest their efforts in the most important 20 %. Session 4 encourages patients to forgive using a self-reflection technique known as the “REACH” intervention, developed by Worthington (Seligman 2003). The patients are instructed to recall hurtful experiences and then to try to understand from the perpetrator’s point of view why this person hurt them. They are asked to recall a time they transgressed, felt guilty, and were forgiven. In return for forgiveness from another person, they might be more inclined to forgive their offender. Session 5 promotes optimism and hopefulness using a sentence-completion technique, devised by the psychotherapist Nathaniel Branden (Ben-Shahar 2007). In this simple technique, patients generate endings to incomplete sentences, helping them to come up with insights that bring about meaningful change in their lives. For instance, the phrase “If I had twice as much ability as I already have, I would become aware…………..” might be completed as follows: “If I had twice as much ability as I already have, I would become aware that I could make others happier by helping them.”

**Table 1:** Content of HSC group psychotherapy

Session (90 min/session)	HSC group psychotherapy
1	Orientation from the view of Spiritual Life Lessons: Causality and Reincarnation
2	Love: Shift from a Love that Takes toward a Love that Gives
3	Wisdom: Shift from Perfectionism toward Optimalism
4	Self-reflection: Shift from Anger, Hatred, and Grudge toward Forgiveness
5	Progress: Shift from Hopelessness toward Hopefulness

For comprehension purposes, participants in the treatment group were provided with a brief booklet and were shown some video films which introduced each session topic. In addition, they were encouraged to carry out homework between sessions. The participants in the delayed-treatment control group continued with their usual medical care including either weekly or monthly case manager and/or psychiatrist visits. They also continued any medication management if applicable. They did not, however, have any group meetings during the 5-week intervention period. After completing the final questionnaires, they received the 5-week intervention as courtesy for participating in the study.

### Statistical Analysis

Descriptive statistics were run on all variables to examine frequency distributions and to identify missing data and outliers. Group comparisons of descriptive data were carried out using *t*-tests for continuous variables (age, education years, BMI, mood status, and illness duration) and Chi-square tests for categorical variables (current smoking and alcohol intake (YES or NO); marital status (single, married, or divorced); religious belief (Buddhism, Shintoism, Christianity, Atheism or Materialism, or impossible to answer); diagnosis (depression [ICD-10 F32-33], panic disorder [ICD-10 F40.01, F41.0], social anxiety disorder (SAD) [ICD-10 F40.1], general anxiety disorder (GAD) [ICD-10 F41.1], obsessive–compulsive disorder (OCD) [ICD-10 F42], somatoform disorder [ICD-10 F45], and eating disorder [ICD-10 F50]); and medication (antidepressant, minor tranquilizer, and/or sleep inducer). Group comparisons of pre-intervention, post-intervention, and 3-month follow-up mean change scores (CES-D score at each time point—CES-D score at pre-intervention) were carried out using repeated analysis of covariance (ANCOVA), controlling for the descriptive characteristic shown in Table 2. The main analysis used total CES-D mean change scores as the outcome, and additional analyses were carried out with mean change scores on the separate CES-D subscales as outcome variables. In each analysis, only the descriptive characteristics which were significantly different between the control and trial groups were controlled in repeated ANCOVA. Sample size calculation was based on the absolute change in depressive symptoms as measured by the CES-D questionnaire from the pre-intervention to the post-intervention and at 3-month follow-up assessments. The trial is powered to detect an effect size of 4.5 or larger on the CES-D in a one-sided test (*α* = 0.05) at a power of 80 % (1-β), and assuming a common standard deviation of the CES-D scores of 8.4, a total of 106 participants (*n* = 53 in each group) are needed in the study. All analyses were performed using Statistical Package for the Social Sciences (SPSS, version 21.0), and all tests of statistical significance were based on two-sided probability.

**Table 2:** Participant characteristics

	Control group (*N* = 60)	Trial group (*N* = 58)
Age, years	36.3 ± 9.5	35.5 ± 10.1
Men, %	46.3	53.7
Education, years	13.9 ± 2.7	14.6 ± 2.1
Single/divorced, %	60.0	60.3
Religion		
Buddhism	18.3	19.0
Shintoism	8.3	5.2
Christianity	1.7	1.7
Atheism or materialism	26.7	29.3
Impossible to answer	45.0	45.8
Current smokers, %	41.7	36.2
Current drinkers, %	61.7	65.5
Body mass index, kg/m^2^	23.3 ± 5.2	23.2 ± 5.3
Diagnosis, %		
Depression	86.7	77.6
Panic disorder	6.7	6.9
SAD	5.0	6.9
GAD	0.0	1.7
OCD	1.7	1.7
Somatoform disorder	0.0	3.4
Eating disorder	0.0	7.1
Illness duration, months.	21.7 ± 31.3	27.7 ± 43.7*
Medication, %		
Antidepressant	86.7	89.7
Minor tranquilizer	43.8	56.3
Sleep inducer	65.0	37.9*
CES-D total score	24.4 ± 11.9	25.5 ± 10.5

Values are mean ± SD, unless indicated otherwise; *CES*-*D* center for epidemiologic studies depression scale, *GAD* general anxiety disorder, *OCD* obsessive–compulsive disorder, *SAD* social anxiety disorder; *p < 0.05 compared to the corresponding value in the control group

## Results

As shown in Table 2, participants in the trial aged around 36 years on average, with roughly equal numbers of men and women. There were no differences in any demographic or diagnostic factors; the majority of patients (97/118 = 82.2 %) were diagnosed with depression. There were two significant differences between groups at baseline. Illness duration was an average 6 months longer in the treatment than in the control group (*p* = 0.03), while fewer treatment group participants were taking sleep medication (*p* = 0.003). Baseline CES-D scores averaged over 24 in both groups, indicating substantial elevations in depressive symptoms.

**Fig. 2:**
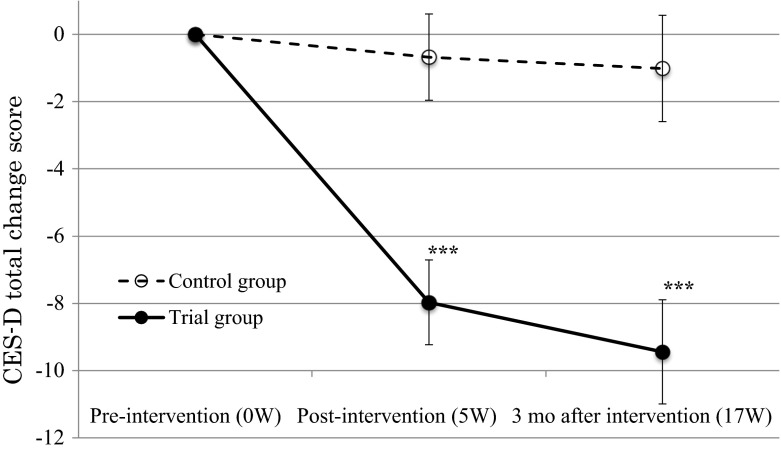
Average total CES-D change scores by group at pre-intervention (0 W), post-intervention (5 W), and 3-month follow-up (17 W). Values are mean ± SE; CES-D, center for epidemiologic studies depression scale; change scores were computed using the following formula (CES-D total score at each time point − CES-D total score at pre-intervention); all analyses controlled for illness duration and taking sleep medication; ***p < 0.001 compared with the corresponding value in the control group

A repeated ANCOVA evaluation (controlling for illness duration and taking sleep medication) on CES-D total score and the four CES-D subscales (depressive affect, positive affect, somatic complaints, and interpersonal difficulties) showed that only somatic complaint scores were significantly lower in the treatment group than in the control group both at post-intervention (5 W) and at 3-month follow-up (17 W) (Table 3). An average total CES-D change score significantly improved at post-intervention (5 W) and was sustained at 3-month follow-up (17 W) (*p* < 0.001) (Fig. 2). Further analyses on the CES-D subscale change scores showed a significant improvement in depressive affect (*p* < 0.001), somatic complaints (*p* < 0.001), and interpersonal difficulties (*p* = 0.007), but not positive affect (*p* = 0.08), both at post-intervention (5 W) and at 3-month follow-up (17 W).

**Table 3:** Comparison of pre-intervention (0 W), post-intervention (5 W), and 3-month follow-up (17 W) mean CES-D change scores by group

	0 W	5 W	17 W	*p*
CES-D total score				
Control group (*N* = 60)	24.2 ± 1.5	23.5 ± 1.5	23.2 ± 1.6	0.05
Treatment group (*N* = 58)	25.6 ± 1.5	17.7 ± 1.5	16.2 ± 1.7	
CES-D depressive affect				
Control group (*N* = 60)	6.8 ± 0.6	6.7 ± 0.6	6.5 ± 0.7	0.26
Treatment group (*N* = 58)	7.9 ± 0.7	4.9 ± 0.6	4.5 ± 0.7	
CES-D positive affect				
Control group (*N* = 60)	4.6 ± 0.4	4.9 ± 0.4	5.1 ± 0.4	0.21
Treatment group (*N* = 58)	4.5 ± 0.4	5.9 ± 0.4	6.1 ± 0.4	
CES-D somatic complaints				
Control group (*N* = 60)	9.5 ± 0.7	9.2 ± 0.7	9.1 ± 0.7	0.01
Treatment group (*N* = 58)	9.6 ± 0.7	6.2 ± 0.7**	5.4 ± 0.7**	
CES-D interpersonal difficulties				
Control group (*N* = 60)	1.3 ± 0.2	1.4 ± 0.2	1.5 ± 0.2	0.54
Treatment group (*N* = 58)	1.7 ± 0.2	1.1 ± 0.2	1.0 ± 0.2	

Values are mean ± SE; *CES*-*D* center for epidemiologic studies depression scale; change scores were computed using the following formula [CES-D subscale score at each time point − CES-D subscale score at pre-intervention]; all analyses controlled for illness duration and taking sleep medication; *p* values were computed using repeated measures ANCOVA models; ** *p* < 0.01 compared to the corresponding value in the control group

As shown in Table 4, the treatment effect on the total CES-D change score was similar in patients diagnosed with depression compared to that for the entire sample (*p* < 0.001). Another subgroup analysis demonstrated that non-religious patients (*p* = 0.003) but not religious patients (*p* = 0.46) had significant decreases in the total CES-D change score at post-intervention (5 W) and at 3-month follow-up (17 W).

**Table 4:** Comparison of pre-intervention (0 W), post-intervention (5 W), and 3-month follow-up (17 W) mean CES-D total change scores by subgroup

	5 W	17 W	*p*
Overall patients			
Control group (*N* = 60)	−0.7 ± 1.3	−1.0 ± 1.6	<0.001
Treatment group (*N* = 58)	−8.0 ± 1.3***	−9.5 ± 1.6***	
Depression patients			
Control group (*N* = 52)	−0.4 ± 1.4	−0.3 ± 1.8	<0.001
Treatment group (*N* = 45)	−8.7 ± 1.5***	−10.0 ± 1.9***	
Religious patients			
Control group (*N* = 17)	−3.4 ± 2.2	−3.4 ± 3.6	0.46
Treatment group (*N* = 15)	−6.1 ± 2.4	−7.3 ± 3.9	
Non-religious patients			
Control group (*N* = 16)	3.1 ± 2.5	2.4 ± 3.4	0.003
Treatment group (*N* = 17)	−9.7 ± 2.5**	−10.1 ± 3.3*	

Values are mean ± SE; *CES*-*D* center for epidemiologic studies depression scale; “religious patients” means those who believe in Buddhism, Shintoism, or Christianity, “non-religious patients” means those who are atheist or materialist; change scores were computed using the following formula [CES-D total score at each time point − CES-D total score at pre-intervention]; all change scores were adjusted for illness duration and taking sleep medication in the analyses of overall effects and depression patients; for education, illness duration, and taking sleep medication in the analysis of religious patients; and for current drinking in the analysis of non-religious patients; *p* values were computed using repeated measures ANCOVA models; * *p* < 0.05, ** *p* < 0.01, and *** *p* < 0.001 compared to the corresponding value in the control group

## Discussion

The current trial has reconfirmed the findings of our previous pilot RCT showing that the HSC group psychotherapy reduced depressive symptoms in Japanese outpatients diagnosed with a mood disorder, neurotic stress-related and somatoform disorder, or eating disorder. Importantly, the intervention effect was sustained at 3-month follow-up. Although the study participants were suffering from various mental disorders (i.e., panic disorder, SAD, GAD, OCD, somatoform disorder, and eating disorder), the subgroup analysis ascertained that the HSC group psychotherapy had a similar treatment effect in patients diagnosed with depression.

In this study, only 27.1 % of participants reported to believe in Buddhism, Shintoism, or Christianity, although there was no significant difference in religious belief between the control and treatment groups. In line with the present data, the Angus Reid survey (2006) found that 24 % of Japanese respondents indicated that religion was very important in their daily lives (Angus Reid 2014), while an average of 48 % of respondents from around the world indicated so. Thus, given that the treatment effect was evident in non-religious but not religious patients, the HSC group psychotherapy might be more effective in Japanese patients than patients from other religious nations (e.g., 63 % of US respondents indicated that religion was very important in their daily lives). However, further research is needed to determine whether there are international differences in the efficacy of the HSC group psychotherapy. It is not clear why only non-religious patients were responsive to the HSC group psychotherapy. One possible mechanism is religious enlightenment, i.e., coming to learn about religious ideas with little previous experience. Another possibility is that religious participants might have had certain conceptions that were inconsistent with those promoted by the HSC psychotherapy, although more than half of the religious participants were Buddhist. In this study, religion was measured only by religious affiliation, but not with a religiosity scale. Follow-up trials should include a measure of religiosity in order to investigate why only non-religious patients were responsive to the HSC group psychotherapy.

Previous religious/spiritual interventions have not reported the effect of treatment on the specific CES-D subscales. In this study, the treatment effect was stronger for somatic complaints than for the other CES-D subscales. If this is a genuine effect, it could be mediated in part via behavioral pathways. For example, religiosity/spirituality has been related to healthier behaviors, including not smoking, exercising, drinking moderately, having lower dietary fat intake, and having better sleep quality (Koenig et al. 2012; Strawbridge et al. 2001). Another potential interpretation is that religiosity/spirituality contributes to reduced somatic complaints by increasing social support (Koenig et al. 2012). In addition, direct physiological pathways might be involved. Religiosity/spirituality might attenuate sympathetic nervous system activity and enhance parasympathetic activation, leading to decreased blood pressure (Gillum and Ingram 2006; Yeager et al. 2006) and inflammatory cytokine levels (Koenig et al. 1997; Lutgendorf et al. 2004). Religiosity/spirituality has also been related to lower circulating cortisol levels or cortisol responsiveness (Carrico et al. 2006; Dedert et al. 2004; Tartaro et al. 2005) and may therefore contribute to reduced somatic complaints. Previous studies have reported an effect of religious/spiritual interventions on various physical conditions including hypertension (Castillo-Richmond et al. 2000), cancer (Carlson et al. 2007; Schneider et al. 2005; Banerjee et al. 2007), rheumatoid arthritis (Matthews et al. 2000), and human immunodeficiency virus (Astin et al. 2006). It would be useful to evaluate whether the HSC group psychotherapy could be used to treat physical conditions.

As described in the introduction, the HSC group psychotherapy is based on Buddhist theory. A similar psychotherapy is the Buddhist mindfulness-based cognitive therapy (MBCT), which was reported to reduce relapse/recurrence of depression in a RCT with depressive patients (Teasdale et al. 2000). The MBCT intervention used meditation and consisted of an initial orientation session followed by eight weekly 2-h group-training sessions, along with daily homework exercises. After the intervention, relapse/recurrence of depression was assessed bimonthly for 1 year. Future trials of the HSC group psychotherapy are needed with longer follow-up duration, and to compare its efficacy with that of non-religious/spiritual psychotherapies (e.g., traditional cognitive-behavioral psychotherapy) and other religious/spiritual psychotherapies such as the MBCT.

### Limitations

As the control group in this trial received only usual care, the effects of the HSC intervention might have been at least partly due to social contact. Future trials should include a control group that involves some group social activity.

## Conclusion

The findings of this study indicate that the Happy Smile Clinic group psychotherapy was of benefit in treating depressive symptoms in a sample of Japanese psychiatric patients.
